# Enhancing transporter activity in heterologous expression systems with SAHA: a 2500‐times more potent and odorless alternative to butyrate

**DOI:** 10.1002/2211-5463.70015

**Published:** 2025-03-07

**Authors:** Svenja Flögel, Maurice Tust, Samira Boussettaoui, Dietmar Fischer, Dirk Gründemann

**Affiliations:** ^1^ Department of Pharmacology, Faculty of Medicine and University Hospital Cologne University of Cologne Germany

**Keywords:** HDAC inhibitors, heterologous expression, mass spectrometry, promotor silencing, SAHA, sodium butyrate, transporter activity, valproic acid

## Abstract

The functional characterization of plasma membrane transport proteins often relies on their heterologous expression in cultured cells. However, some transporters exhibit low activity, hindering meaningful functional assays. Heterologous expression is usually based on strong viral promoters which in living cells are prone to promoter silencing, a major problem. Here, we investigated the efficacy of low‐cost histone deacetylase (HDAC) inhibitors in enhancing transporter activity, comparing the established sodium butyrate (the sodium salt of butyric acid) with valproate/valproic acid (VPA) and suberoylanilide hydroxamic acid (SAHA, also known as vorinostat). Using 293 cells stably transfected with pEBTet plasmids containing the CMV promotor to express the transporters SLC16A9, SLC22A15, and OATP1A2, we measured substrate efflux or uptake via LC–MS/MS following overnight preincubation with the HDAC inhibitors. All three compounds markedly stimulated transporter activity. VPA was less effective than butyrate but still surpassed control conditions. SAHA was cytotoxic at 6 μm, but at 2 μm, the enhancement was consistently comparable to 5 mm butyrate. Additionally, SAHA was more cost‐effective and devoid of the repulsive odor characteristic of butyrate. Our findings advocate for replacing butyrate with SAHA to enhance heterologously expressed transporter activity. This offers a more efficient and user‐friendly alternative for functional assays.

## Graphical abstract

Heterologous expression of membrane transporters in cultured cells is essential for functional characterization, but is sometimes limited by low activity. Our study compares the HDAC inhibitors butyrate, VPA and SAHA to enhance transport activity. We propose to replace butyrate by SAHA: it is equally effective, devoid of repulsive odor, costs less, and the required concentration is 2500 times lower.

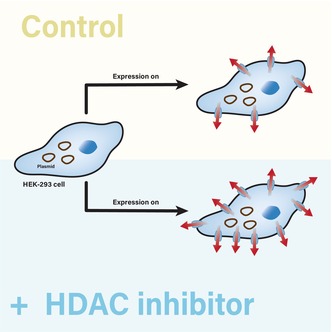

**Abbreviations**
E3S: estrone‐3‐sulfate
LC: liquid chromatography
MS: mass spectrometry
SAHA: suberoylanilide hydroxamic acid
VPA: valproic acid

The functional characterization of transport proteins of the plasma membrane is largely based on heterologous expression in cultured cells. This involves measuring the uptake of substrates into the cells or their release from the cells. Unfortunately, it happens that the activity of some transporters is only very low, perhaps too low for a meaningful measurement of function. In this case, there is at least a chance of boosting the transport activity by an overnight preincubation of the transfected cells with 5 mm of the sodium salt of butyric acid (sodium butyrate). Butyrate has numerous effects on cultured mammalian cells including inhibition of proliferation, changes in morphology and cytoskeleton, induction of differentiation, and activation or inhibition of gene expression [1, 2, 3].

Butyrate reportedly acts by inhibiting histone deacetylases (HDACs) [1]. HDACs are enzymes that remove acetyl groups from N‐ε‐acetyl lysine in histones (and also other proteins). Reversible acetylation occurs on specific lysines located in the N‐terminal tails of the core histones; these tails protrude from the nucleosome (= histone octamer). Each nucleosome has at least 28 potential sites of acetylation [1]. Positive charges on the lysines promote chromatin condensation; the genomic DNA is wrapped (146 bp per nucleosome) more tightly around the histones, compacting the chromatin structure. Thus, HDAC activity results in transcriptional repression, whereas histone acetyltransferases activate transcription by opening the chromatin. The acetylation and deacetylation of histones is a powerful form of epigenetic regulation of gene expression.

HDAC inhibitors promise to stimulate gene expression. Several classes of inhibitors are available, like short‐chain fatty acids [as sodium butyrate and valproate (VPA, valproic acid; Fig. 1)], epoxides (as depudecin and trapoxin), cyclic peptides, benzamides, and hydroxamic acids [as trichostatin A and suberoylanilide hydroxamic acid (SAHA; INN vorinostat)] [4]. It is important to note the complexity of HDAC inhibition, since higher eukaryotes can express a total of 11 “classical” enzymes (HDAC1 to HDAC11). Although isoform‐selective inhibitors have been developed, most compounds inhibit several enzymes in parallel. Moreover, HDAC inhibitors can also alter the degree of non‐histone protein acetylation and thereby increase or repress activity. In particular, they have been shown to alter the activity of many transcription factors [5].

**Fig. 1:**
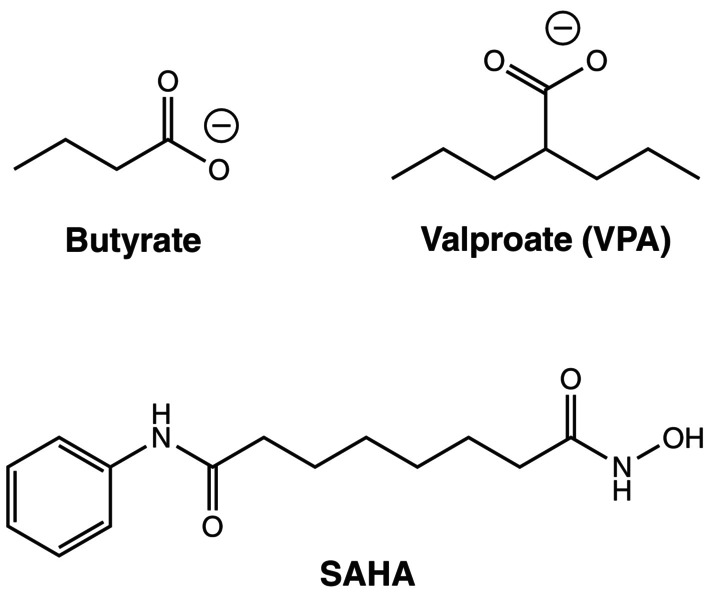
Compound structures.

The exact cascade of events by which butyrate enhances the activity of some transporters when expressed from plasmids or other vectors in cultured cells is unclear. If the effect is indeed based on the inhibition of deacetylation, it might be worthwhile to compare butyrate with other HDAC inhibitors. We have not found such a comparison in the literature. In this study, we compared two widely available, low‐price inhibitors, VPA and SAHA, with butyrate by measuring the activity of three different transporters. SAHA binds directly to the catalytic site of the HDACs thereby blocking substrate access [6]. It inhibits HDACs at 50–200 nm (IC_50_ values) and arrests cell growth in a wide variety of transformed cells in culture at 2.5–5.0 μm.

Our results presented here suggest to move from butyrate to SAHA: SAHA has virtually the same stimulating effect, but the required concentration is 2500 times lower — and it is devoid of the repulsive butyrate odor.

## Methods

### Plasmid constructs

All transporter cDNAs were expressed from the vector pEBTetLNC, an Epstein–Barr plasmid vector expressing the tetracycline repressor to allow inducible protein expression in human cell lines [7, 8].

The plasmids expressing SLC22A15 from human and SLC16A9 from human have been described previously [9]. The amino acid sequence of the open reading frame of mouse SLC22A15 cDNA matches the GenBank accession number NM_001039371. In pEBTetLNC/SLC22A15m, the 5′‐interface between the vector and the transporter cDNA is **CGTTT AAACTT AAGCTT GGTACC GAGCTC GGATCC** gccacc ATG GAG GTG GAG GAG GCG TTC CAG G (polylinker in bold, Kozak sequence in lowercase, start codon underlined). The 3′‐interface is GAT GAG GAG ACC CAG ATG ATC AAG TGA **GCGGCCGC GGGGCA GTGCAT GTAATC** (cDNA underlined, polylinker in bold).

The amino acid sequence of the open reading frame of human OATP1A2 cDNA (gene symbol *SLCO1A2*) matches the GenBank accession number NM_134431. In pEBTetLNC/OATP1A2h, the 5′‐interface between the vector and the transporter cDNA is **CGTTT AAACTT GGTACC** gccacc ATG GGA GAA ACT GAG AAA AGA ATT. The 3′‐interface is GAT GAT GAA TTG AAA ACT AAA TTG TAA **GCGGCCGC GGGGCA GTGCAT GTAATC**.

### Cell culture

293 cells (ATCC CRL‐1573, also referred to as HEK‐293 cells) were cultured as detailed previously [9]. Stably transfected cell lines were generated as reported before [7], using Turbofect (R0531, Thermo Fisher Scientific, Dreieich, Germany). Due to the episomal propagation of pEBTet‐derived vectors, cell pools were used instead of single clones. To ensure plasmid permanence, the growth medium always contained 3 μg·mL^−1^ puromycin (13 884, Cayman Chemical, Ann Arbor, MI, USA). To switch on transporter expression, cells were incubated for at least 20 h with 1 μg·mL^−1^ doxycycline (195 044, MP Biomedicals, Eschwege, Germany). Paired control cells (expression off, no addition of doxycycline) were from the exact same maintenance culture.

### Transporter assays

Transporter assays were carried out with stably transfected 293 cells. Cells were seeded in 60 mm polystyrol dishes (83.3901, Sarstedt, Nümbrecht, Germany) coated with 0.1 g·L^−1^ poly‐L‐ornithine (P3655, Merck, Darmstadt, Germany) in 0.15 m boric acid pH 8.4 and grown to reach a confluence of ca. 80%. Some cells were preloaded overnight with creatine (27910, Merck) to increase intracellular concentrations. KRH buffer contained 125 mm NaCl, 25 mm HEPES‐NaOH pH 7.4, 5.6 mm (+)glucose, 4.8 mm KCl, 1.2 mm KH_2_PO_4_, 1.2 mm CaCl_2_, and 1.2 mm MgSO_4_; phosphate was omitted in efflux assays to reduce contamination of the MS instrument. HBSS buffer contained 140 mm NaCl, 5.6 mm (+)glucose, 5 mm KCl, 4.0 mm NaHCO_3_, 1.0 mm CaCl_2_, 0.5 mm MgCl_2_, 0.4 mm MgSO_4_, 0.3 mm Na_2_HPO_4_, and 0.3 mm KH_2_PO_4_. Due to the low buffering capacity of HBSS, phosphate was not omitted for efflux experiments.

For uptake experiments, cells were washed 2–3 times with 4 mL of warm KRH or HBSS which was then replaced by 2 mL of buffer containing the substrate of interest. Cells were incubated for a defined time on a water bath at 37 °C. The uptake was stopped by washing three times with 4 mL of ice‐cold buffer. Cells were then lysed with 1 mL methanol for at least 20 min. The samples were stored at −20 °C until LC–MS measurement.

For efflux experiments, cells were washed three times with 4 mL of warm efflux buffer (KRH or HBSS) which was then replaced by 2 mL of efflux buffer. Cells were incubated for 30 min on a water bath at 37 °C; samples (0.2 or 0.5 mL) were collected from the supernatant at 30 min.

The protein content was determined from three paired dishes lysed with 1 mL 0.1% v/v Triton‐X 100 in 5 mm Tris–HCl pH 7.4. The bicinchoninic acid assay (Pierce; Thermo Scientific 23225, Life Technologies, Darmstadt, Germany) was used with bovine serum albumin as standard.

### LC–MS/MS

The substrates were measured by LC–MS/MS as described previously for creatine, D3‐creatine [9], and estrone‐3‐sulfate [10].

### Calculations and statistics

Assays were at least performed with three replicates per condition as shown in the figures. Some results are given as arithmetic mean ± SEM (*n* ≥ 3). Graphs were created using graphpad prism (version 10.3.1, GraphPad Software, San Diego, CA, USA). Statistical significance was determined with unpaired *t*‐tests, using a significance level of 0.05 for a comparison of two groups; two‐tailed *P*‐values are given.

### Materials

If not stated otherwise, compounds were obtained from Merck (formerly Sigma‐Aldrich, Munich, Germany). SAHA (10009929, Cayman Chemical), sodium butyrate (8.17500), valproic acid sodium salt (P4543).

## Results

The function of transport proteins was assayed with 293 cells that were stably transfected with pEBTetLNC plasmids carrying transporter cDNAs. pEBTetLNC, like pEBTetD, is an Epstein–Barr replication plasmid for doxycycline‐inducible protein expression in human cell lines based on the simple tetracycline repressor [7, 8]. Cells were grown in dishes to nearly confluent monolayers. By adding 1 μg·mL^−1^ doxycycline to the culture medium overnight, the expression of transporters was switched on; in the paired control cells, expression remained off. After thorough washing, the uptake or efflux of substrates was assayed as detailed in the figure legends. The solute content of cell lysates or supernatants was measured by LC–MS/MS.

The HDAC inhibitor butyrate is typically used at 5 mm to enhance transport activity [11]. VPA and SAHA were chosen as potential alternatives, as both are widely available commercially at relatively low cost. Following the literature on the limit of tolerable toxicity for cultured cells, we chose 2 mm for VPA [12, 13, 14] and 6 μm for SAHA [15]. To examine saturation, each compound was also tested at a threefold lower concentration. The HDAC inhibitors were added to the overnight incubations (about 20 h) just before the functional assays.

**Fig. 2:**
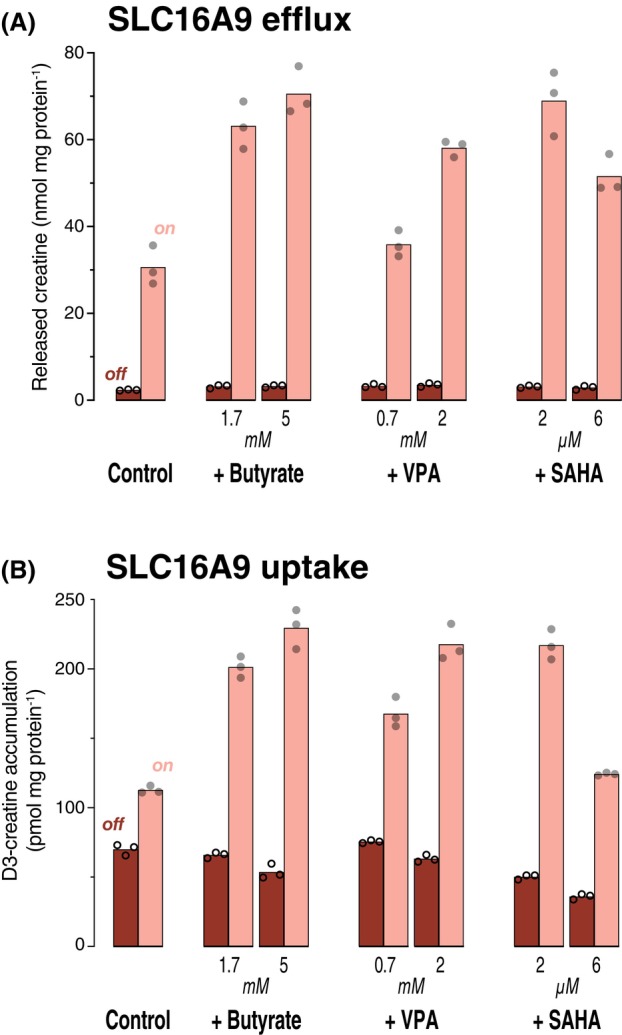
Stimulation of SLC16A9 transport activity. (A) 293 cells expressing SLC16A9 from human (on) and paired control cells (expression off) were preincubated overnight with 10 mm creatine (on) or 1 mm creatine (off) plus butyrate, VPA, or SAHA as stated. For the efflux assay, cells were washed and incubated at 37 °C in KRH without phosphate. After 30 min, samples were collected from the supernatant to determine the creatine efflux via LC–MS/MS. Each circle represents one dish (*n* = 3). (B) 293 cells expressing SLC16A9 from human (on) and control cells (expression off) were preincubated overnight with butyrate, VPA, or SAHA as stated. In the uptake assay, the cells were incubated for 4 min at 37 °C with 10 μm D3‐creatine in KRH buffer. After washing and lysis of the cells, the substrate accumulation in the cell lysates was determined by LC–MS/MS. Each circle represents one dish (*n* = 3); bars indicate the arithmetic mean.

The efflux of creatine via SLC16A9 was clearly stimulated after preincubation with 5 mm butyrate (Fig. 2A; unpaired *t*‐test, *P* < 0.001); the transporter‐mediated efflux (= on minus off) was 28 ± 3 nmol mg protein^−1^ for control cells and 67 ± 3 nmol mg protein^−1^ for 5 mm butyrate. VPA was less effective, but 2 μm SAHA stimulated to the same level as 5 mm butyrate (66 ± 4 nmol mg protein^−1^; *P* = 0.8). The decrease of efflux at 6 μm relative to 2 μm SAHA was caused by visible toxicity, leading to cell death. The comparison of efflux could be affected by intracellular creatine concentration differences resulting from pre‐assay transporter activity. We therefore also measured the uptake of deuterium‐labeled creatine into cells via SLC16A9. The results were very similar to the efflux assays (Fig. 2B).

**Fig. 3:**
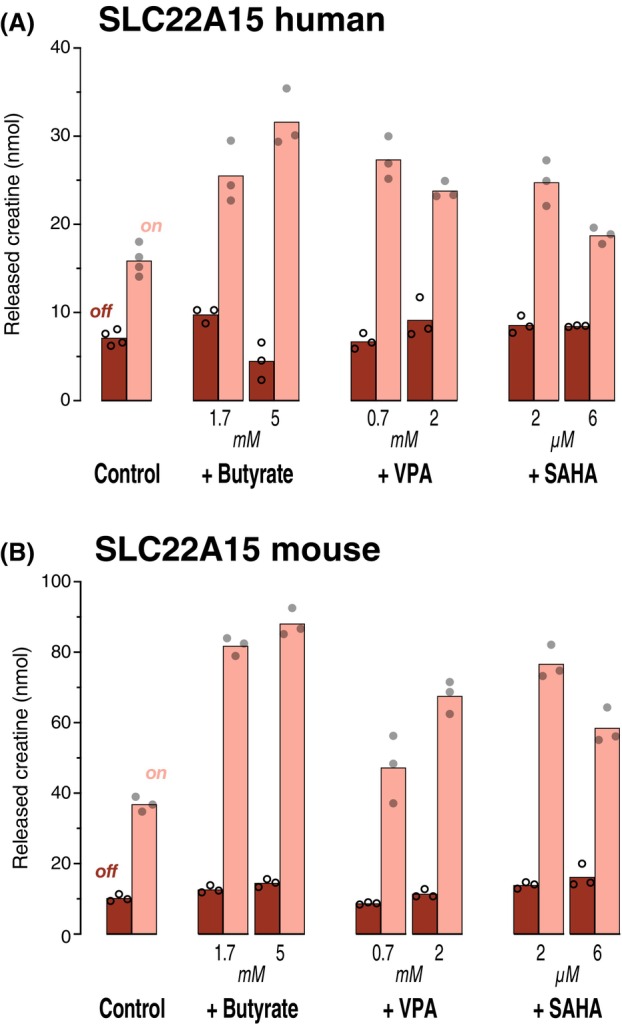
Stimulation of SLC22A15 transport activity. 293 cells expressing (on) either SLC22A15 from human (A) or SLC22A15 from mouse (B) and paired control cells (expression off) were preincubated overnight with 1 mm creatine plus butyrate, VPA, or SAHA as stated. For the efflux assay, cells were washed and incubated at 37 °C in HBSS buffer plus 5 mm guanidinoacetic acid to drive creatine efflux [9]. After 30 min, samples were collected from the supernatant to determine the creatine efflux via LC–MS/MS. Each circle represents one dish (*n* = 3); bars indicate the arithmetic mean.

The efflux of creatine via SLC22A15 [9, 16] from human or mouse was also stimulated after preincubation with 5 mm butyrate (Fig. 3). The mouse transporter effects were very similar to the SLC16A9 data. Cells expressing human SLC22A15 appeared impaired under the microscope after incubation in 2 mm VPA or 6 μm SAHA; here, 2 μm SAHA was slightly less stimulating than 5 mm butyrate (*P* = 0.01).

**Fig. 4:**
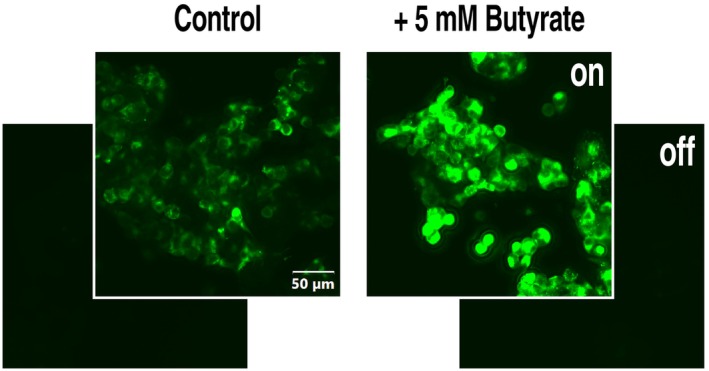
Butyrate preincubation enhances SLC22A15 protein expression. 293 cells were transfected with a pEBTetLNC plasmid carrying SLC22A15h with an eGFP tag at the C‐terminus and seeded on microscope slides. After overnight incubation in cell culture medium with or without 5 mm butyrate and concurrently with (“on”) or without doxycycline (“off”) to regulate protein expression, cells were fixed with paraformaldehyde, embedded in Mowiol, and imaged using a Slideview VS200 microscope (Olympus, Hamburg, Germany) at 20× magnification with an exposure time of 43 ms. eGFP fluorescence is shown in green.

In a control experiment with eGFP‐tagged SLC22A15, preincubation with 5 mm butyrate clearly increased the fluorescence signal (Fig. 4). This suggests that butyrate increases the amount of protein rather than altering trafficking or protein modification.

**Fig. 5:**
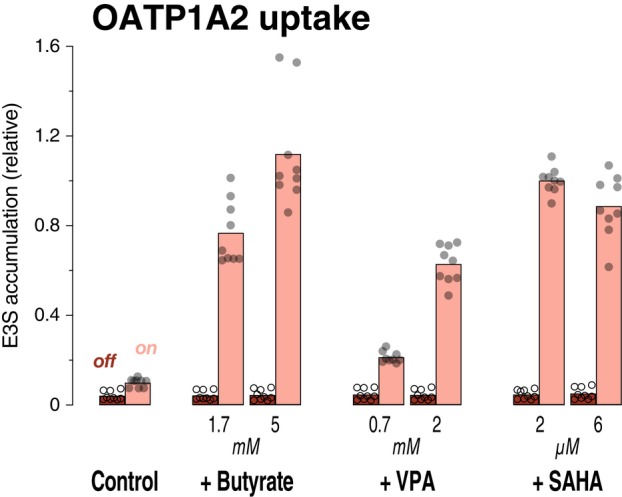
Stimulation of OATP1A2 transport activity. 293 cells expressing OATP1A2 from human (expression on) and paired control cells (off) were preincubated overnight with butyrate, VPA, or SAHA as stated. In the uptake assay, the cells were incubated for 7 min at 37 °C with 10 μm estrone‐3 sulfate (E3S) in KRH buffer. After washing and lysis of the cells, the substrate accumulation in the cell lysates was determined by LC–MS/MS. Each circle represents one dish; bars indicate the arithmetic mean. To demonstrate the robustness and reliability of our results, this experiment was performed three times, on different days, with different maintenance cultures. Since the absolute uptake varied across the experiments, for each experiment (*n* = 3), the accumulation of E3S was expressed relative to the mean of the 2 μm SAHA data (expression on) here.

The accumulation of E3S via OATP1A2 was strikingly stimulated after preincubation with 5 mm butyrate (Fig. 5; unpaired *t*‐test, *n* = 9; *P* < 0.0001); the transporter‐mediated uptake (= on minus off, relative to 2 μm SAHA total uptake) was 0.06 ± 0.01 for control cells and 1.08 ± 0.08 for 5 mm butyrate. Again, VPA was less effective, but 2 μm SAHA stimulated to a level very similar to 5 mm butyrate (0.95 ± 0.02; *P* = 0.15).

## Discussion

The activity of some transporters, when expressed heterologously in cultured cells, for example from transfected plasmids, can be markedly enhanced by preincubation with 5 mm butyrate. In this study, the transporters SLC16A9, SLC22A15 and OATP1A2 were examined, which respond well to butyrate. However, there are also transporters whose activity in our expression system is not altered by butyrate, for example, SLC16A12. Since even moderate overdosing results in cell damage for all inhibitors, it is important to determine the optimal concentration and incubation time for each compound and cell line. Maximum bulk histone acetylation appeared 12–16 h after adding VPA [12]. In our experiments, preincubations > 24 h were not advantageous; in three‐day‐incubations we observed extensive cell death and damage (not shown).

Our data show that the HDAC inhibitors VPA and SAHA consistently have effects similar to butyrate. SAHA was generally as effective as butyrate, whereas VPA was less effective. SAHA has the advantages over butyrate of the absence of disgusting odor and a lower dosage by a factor of 2500 (2 vs. 5000 μm) which implies fewer unwanted effects. Indeed, butyrate inhibits HDACs but also has several other activities [2]; recently, butyrate at 1 mm was reported to modulate the serotonin uptake transporter SERT [17]. In addition, the price of SAHA per application was 2.4 times lower: 1 g SAHA (Mr = 264.3) costs 249 EUR (Cayman Chemical via Biomol, Hamburg, Germany); 1 L at 2 μm requires 0.53 mg equivalent to 13 Cent. 250 g sodium butyrate (Mr = 110.1) costs 139 EUR (Cayman Chemical via Biomol); 1 L at 5 mm requires 0.55 g equivalent to 31 Cent. Note that other HDAC inhibitors are much more expensive; at the lower price end, 10 mg trichostatin A, for example, is available for ca. 600 EUR; it is used at concentrations around 1 μm. We therefore recommend replacing butyrate with SAHA to activate heterologously expressed transporters.

The exact mechanisms by which the compounds work are unclear. Butyrate has numerous effects on cultured mammalian cells including inhibition of proliferation, induction of differentiation and induction or repression of gene expression [18]. It is evident that butyrate initiates a highly complex reprogramming of the cell: > 7% of all genes examined exhibited *sustained* alterations in expression beyond levels defined by untreated control cells [19]. As a minimal estimate of the complexity of the response to butyrate, at least 1000 genes, out of approximately 10 000–15 000 per cell, showed substantial alterations in expression [19]. In other studies with trichostatin A and SAHA, ~ 2% of cellular genes (8 genes out of 340 examined) [20] or less than 2% [21] were affected.

It seems sensible to distinguish between endogenous and heterologous gene expression. Butyrate can increase (MDR1 [22]) or inhibit (MRP1 [23]) the expression of endogenous transporters in cell lines. Butyrate did not affect the endogenous transporter MRP2 [22], but the heterologous expression of MRP2 was strongly increased in other cells [11]. In promoters of butyrate‐responsive genes, the action of butyrate is often mediated through Sp1/Sp3 binding sites [1]. In contrast, heterologous expression is usually based on strong viral promoters which are prone to promoter silencing, a major problem, with transgenes driven by tetracycline‐regulated promoters [24], but also *in vivo* [25]. It has been proposed that HDAC inhibitors such as butyrate and trichostatin A open the chromatin structure of genome‐integrated viral sequences and thereby activate transcription [26]. Indeed, after the insertion of plasmid DNA into the genome and butyrate preincubation, there was a marked increase in transporter protein in Western blots using crude membrane fractions and membrane vesicles [11]. Our results with eGFP‐tagged SLC22A15 (Fig. 4) also indicate increased protein expression. It seems unlikely that HDAC inhibitors stimulate transporter modification (e.g. phosphorylation) or trafficking to the plasma membrane to account for the enhanced transport activity.

Our EBNA1/*oriP*‐based plasmids are maintained as a stable episome, that is, extrachromosomal plasmid copies in the nucleus; with similar plasmids, 293 cells contained 117 ± 1 copies per cell [27]. In the nucleus, the plasmids, like genomic DNA, associate with histones to form nucleosomes [28]. With our pEBTet plasmids, using the CMV promoter for transgene expression, we only see activation for some transporters, but no effect for others; never have we observed inhibition by butyrate. It may be that some transporter cDNAs, depending on their nucleotide sequence, immediately cause promoter silencing by binding tightly to histones, while others bind less tightly. However, other features, like the number of CpG sites, contribute to epigenetic silencing of the transgene carried by the episome [29]. Further research is needed to better understand the pathways involved and to refine the use of compounds in optimizing transporter expression or activity.

In conclusion, our study compares the HDAC inhibitors butyrate, VPA and SAHA to enhance the activity of specific transporters expressed heterologously in cultured cells. We propose to replace butyrate by SAHA: it is equally effective, devoid of repulsive odor, costs less, and the required concentration is 2500 times lower.

## Conflict of interest

The authors declare no conflict of interest.

### Peer review

The peer review history for this article is available at https://www.webofscience.com/api/gateway/wos/peer‐review/10.1002/2211‐5463.70015.

## Author contributions

DG, SF, MT, and SB planned experiments. SF, MT and SB performed experiments. DG, MT and SF analyzed data. DG, SF, and MT wrote the article. DF provided resources. All authors reviewed and approved the final article version.

## Data Availability

The data that support the findings of this study are available in the figures of this article. Data not shown are available from the corresponding author (dirk.gruendemann@uni-koeln.de) upon reasonable request.
